# OLDER PEOPLE LIVING WITH HIV: THE RELATIONSHIP BETWEEN COMMUNITY-LEVEL FACTORS AND THEIR HIV HEALTH

**DOI:** 10.1093/geroni/igz038.1182

**Published:** 2019-11-08

**Authors:** Yookyong Lee, Rick Walton, Edward Jackson, Lindsey Jackson, Scott Batey

**Affiliations:** 1 University of Alabama at Birmingham, Birmingham, Alabama, United States; 2 NHS CSAB, Birmingham, Alabama, United States; 3 University of Alabama at Birmingham Center for AIDS Research, Birmingham, Alabama, United States; 4 Birmingham AIDS Outreach/Magic City Wellness Center, Birmingham, Alabama, United States

## Abstract

According to Centers for Disease Control and Prevention, older adults (aged 50 and older) accounted for 17% of new HIV diagnoses in 2016. The number of older people living with HIV (PLWH) is increasing because of antiretroviral therapy that allows a near normal life expectancy with HIV. Consequently, older PLWH are more likely to develop diseases associated with aging and be affected by polymedication. However, their lived experience and challenges that they face have been underexamined. In this study, we focused on community-level factors, which have potential health implications for older PLWH. We examined a subsample of participants who were at least 50 years-old (n=20; Mean=55; range 50–60) from a community-based participatory research study (n=40). Participants, who previously established HIV care, were recruited through word-of-mouth and flyers posted in the community. Interviews were recorded, transcribed, coded, and triangulated through an iterative process during team meetings. In this subsample (n=20), majority of participants were male (75%) and Black (80%). Older PLWH reported crime, social isolation (interacting with crime and stigma), and lack of access to resources (e.g., transportation, grocery store, pharmacy) as factors negatively associated with their physical and emotional health. A more holistic, complex approach to support older PLWH is essential. They should experience a greater quality of life along with longevity. Therefore, more attention to contextual and structural barriers associated with treatment and care is necessary to find a way for higher retention, (re)engagement, and adherence to care among older PLWH.

